# Perspectives of older people engaging in nurse-led cardiovascular prevention programmes: a qualitative study in primary care in the Netherlands

**DOI:** 10.3399/bjgp15X683149

**Published:** 2014-12-29

**Authors:** Suzanne A Ligthart, Karin DM van den Eerenbeemt, Jeanette Pols, Emma F van Bussel, Edo Richard, Eric P Moll van Charante

**Affiliations:** Department of Medical Ethics; Department of Medical Ethics; Department of Medical Ethics; Department of Neurology; Department of Neurology; Department of General Practice, Academic Medical Center, University of Amsterdam, the Netherlands.

**Keywords:** cardiovascular diseases, older people, prevention, primary health care, qualitative research

## Abstract

**Background:**

Cardiovascular prevention programmes are increasingly being offered to older people. To achieve the proposed benefits, adherence is crucial. Understanding the reasons for adherence and non-adherence can improve preventive care.

**Aim:**

To gain insight into what motivates older people living in the community to partake in a cardiovascular prevention programme, and reasons for subsequent continuation or withdrawal.

**Design and setting:**

Qualitative study of current and former participants of the ongoing ≥6 year PreDIVA (prevention of dementia by intensive vascular care) trial in primary care practices in suburban areas in the Netherlands.

**Method:**

Semi-structured interviews were conducted with a purposive sample of 15 participants (aged 76–82 years). Interviews were audiorecorded and analysed by two independent researchers using a thematic approach. Participants were asked about their motivation for participating in the programme, along with the facilitators and barriers to continue doing so.

**Results:**

Responders reported that regular check-ups offered a feeling of safety, control, or being looked after, and were an important motivator for participation. For successful continuation, a personal relationship with the nurse and a coaching approach were both essential; the lack of these, along with frequent changes of nursing staff, were considered to be barriers. Participants considered general preventive advice unnecessary or patronising, but practical support was appreciated.

**Conclusion:**

To successfully engage older people in long-term, preventive consultations, the approach of the healthcare provider is crucial. Key elements are to offer regular check-ups, use a coaching approach and to build a personal relationship with the patient.

## INTRODUCTION

Improvement of the cardiovascular risk profile of older people is projected to have substantial beneficial effects on the incidence of cardiovascular diseases, disability, and possibly also on dementia.^1^–^7^ However, adherence to cardiovascular risk-management guidelines is low, even in secondary prevention settings, and especially among older people.^1^,^8^,^9^ In response to this, over the past decade, various cardiovascular prevention programmes have emerged for the growing population of older people.

Understanding older peoples’ views and experiences regarding participation in preventive interventions can lead to a more patient-centred approach to care and, therefore, better adherence. It has already been shown that providing information on a healthy lifestyle is insufficient to change lifestyle behaviours.^10^ A lack of tailored discussions, scheduled follow-ups, and inadequate timing also appear to be major barriers for satisfactory counselling.^11^–^13^

The ongoing PreDIVA (prevention of dementia by intensive vascular care) trial aims to demonstrate a reduction in the incidence of dementia and disability through nurse-led intensive vascular care.^14^ This programme involves long-term, preventive consultations and comprises a tailor-made approach; it, therefore, generates an excellent opportunity to evaluate the impact of this type of intervention.

The aim of this study was to explore what motivates older people to participate in long-term, nurse-led, preventive cardiovascular consultations in primary care, and what reasons they have for continuing with, or withdrawing from, the programme.

## METHOD

### Participants

This interview study was conducted with a sample of PreDIVA participants. Details of the PreDIVA trial have been published elsewhere^14^ but, in brief, it is an ongoing cluster randomised controlled trial with ≥6 years of follow-up (completion scheduled in March 2015), in which all people aged 70–78 years were invited to participate by a letter from their GP. Those with dementia or conditions likely to hinder successful follow-up were excluded (13%) (for example, language barrier, severe psychiatric disease, or life expectancy <2 years). After baseline assessments (*n* = 3533, 53% of invited population), primary care practices were randomised to standard care or intensive vascular care. Intensive vascular care comprised 4-monthly, nurse-led consultations aimed at reducing cardiovascular risk factors. These consultations included providing cardiovascular medication and lifestyle advice, based on national guidelines; details of the individual components and interventions are given in Table 1. If indicated, medication was started or adjusted in close consultation with the GP.

**Table 1.** Components of intensive vascular care, carried out by nurses during 4-monthly visits

**Component**	**Intervention**
Blood pressure	Advice, medication when needed
Smoking	Counselling, smoking-cessation advice
Exercise	Advice, referral to exercise programme
Weight	BMI>25: advice; BMI >30: referral to dietician
Cholesterol	Advice, medication when needed
Glucose	Stepwise protocol according to guidelines
Alcohol	Advice, referral when problematic

BMI = body mass index.

To meet the aims of this study, only participants who took part in the 4-monthly visits with the nurse were interviewed. Of those approached for an interview, one couple (irritated by changes in staff) and one individual (no reason given) did not want to be interviewed. Participants were purposively sampled to ensure diversity of the following variables:

- sex;
- educational level;
- living arrangements (living alone or with others);
- cardiovascular history;
- number of modifiable risk factors (<2 or ≥2); and
- ongoing, versus discontinued, participation in the PreDIVA trial.

**How this fits in**

Adherence to cardiovascular risk management guidelines is low, especially among older people. Better adherence could result in prevention of cardiovascular disease and related disability, and possibly also dementia. As a result, cardiovascular prevention programmes are increasingly being offered. Older peoples' motivation to take part in such a programme was primarily the possibility to get free regular check-ups. For continued participation, a personal relationship with the nurse appeared to be crucial. The traditional approach of giving general advice was regarded useless or even patronising. Instead, participants were most satisfied and open to change when nurses used a coaching approach.

Ethnicity was not taken into account as 98% of participants were white. All interviewees had participated in the PreDIVA study for at least 4 years, allowing for an in-depth evaluation of the intervention. At the 4-year follow-up measurement of PreDIVA, 66% of participants were still partaking in it. In the intervention group, 18% had stopped at their own request; in the control group this figure was 15% (drop-outs are actively retrieved for final assessment).

### Procedure

The interviews (*n* = 15) for the study took place between September and November 2013, and were semi-structured, with use of a topic list. Participants were encouraged to explore their experiences in terms of the following domains:

- motivation to participate in the study; and
- barriers and facilitators to continue with the study.

The topic list was modified when new themes emerged from the data. The interviews were conducted by two researchers in participants’ home and were audiorecorded. The interviews started with the question: ‘What was your experience of participating in the study?’, followed by more in-depth questioning.

In five interviews, a family member was present at some point — on request of the interviewee — (two daughters, three partners) and provided an occasional comment or answer to illustrate matters that participants found difficult to remember (for example, reason for participation). The duration of the interviews ranged from 33 to 77 minutes. Once data saturation was achieved, after 15 interviews, no additional participants were recruited.

### Analysis

A qualitative thematic analysis was performed, following six phases set out by Braun and Clarke:^15^

- Both interviewers listened to, and read, the material, to become accustomed to the interviews. The first three interviews were transcribed verbatim. They then listened carefully to the audiorecordings of the other 12 interviews, and sentences or paragraphs that were relevant to the targeted domains were transcribed.
- The interviewers independently generated initial codes and systematically collected data relevant to each of the areas of interest.
- Codes were collated into potential themes. For each interview, the independent analyses were merged into a consensus document.
- An in-depth exploration of the detailed analysis was undertaken, generating a thematic ‘map’ of main themes and subthemes. Results were regularly compared and discussed.
- Themes were refined and the narrative of the analysis was proposed.
- Illustrative extracts were selected and data was reported relating the final analysis to the research question and existing literature.

## RESULTS

In total, 15 individuals aged 76–82 years were interviewed from six different healthcare centres. Of those 15, 11 were still participating in the programme; four had withdrawn from the study after some years of participation. Table 2 outlines the participants’ characteristics.

**Table 2.** Participants’ characteristics

**Participant number**	**Age, years**	**Sex**	**Highest education level**	**History of CVD**	**Treatable risk factors^a^**	**Living situation**	**Current participation (duration, years)**
1	77	Female	Vocational training	No	3	Daughter	Ongoing (6)
2	77	Female	Vocational training	No (DM2)	3	Alone	Ongoing (6)
3	77	Female	Vocational training	No	1	Partner	Ongoing (6)
4	76	Male	Higher (vocational) education	Yes	0	Partner	Ongoing (6)
5	78	Female	Higher (vocational) education	No	1	Partner	Ongoing (6)
6	82	Male	Higher (vocational) education	Yes	3	Partner	Discontinued (5)
7	77	Male	Higher (vocational) education	No	1	Alone	Discontinued (4)
8	76	Female	Vocational training	No (DM2)	1	Partner	Ongoing (6)
9	77	Male	Vocational training	Yes	1	Partner	Ongoing (6)
10	80	Female	Elementary school	No	3	Partner	Ongoing (6)
11	82	Male	Elementary school	No (DM2)	0	Partner	Ongoing (6)
12	81	Female	Vocational training	No	2	Alone	Discontinued (5)
13	79	Female	Elementary school	Yes	4	Partner	Discontinued (5)
14	78	Male	Vocational training	Yes	3	Alone	Ongoing (6)
15	81	Female	Vocational training	No	1	Son	Ongoing (6)

CVD = cardiovascular disease. DM2 = type II diabetes mellitus.

a *Treatable risk factors at baseline: hypertension (systolic blood pressure* ≥*160 mmHg), hyperlipidaemia (total cholesterol* ≥*5 mmol/L (primary prevention) or low-density lipoprotein* ≥*2.5 mmol/L (secondary prevention), body mass index:* ≥*30, current smoker (yes/no), inactivity (yes/no).*

### Motivators to participation

The perception of being ‘checked up on’ was an important reason for participation. Many liked to know whether they were doing well with regards to their physical and mental health, and hoped for reassurance and normal test results:

*‘We* [my wife and I] *expected that we would find out how we were doing, intellectually* [as well as physically]*. I was rather interested in that. I wanted to know how I was doing.’*

(P9, male, 77 years, living with partner, ongoing participation)

The check-up as a whole, and laboratory checks and blood pressure in particular, were regarded as useful:

*‘Your blood pressure is measured and once in a while she checks the blood.* […] *Normally, when you don’t have any complaints, you don’t visit the doctor, right? It feels unnecessary. So I’m glad we have this now, it makes me feel very safe.’*

(P5, female, 78 years, living with partner, ongoing participation)

The same was mentioned for evaluation of weight, cognition, and physical activity.

Some decided to participate because they were asked (by their GP) and saw no reason to decline the invitation:

‘I thought it’s probably something good. So why shouldn’t I do it? At the time I was some years younger, and it seemed like a good thing to do. There’s no harm in trying.’

(P10, female, 80 years, living with partner, ongoing participation)

Good accessibility facilitated participation. The programme did not seem to cause fear among participants, but rather offered reassurance that things remained well and they were being cared for.

It seemed that, for many participants, evaluation of their health status was the main reason for partaking, instead of actively making changes themselves. They also neither participated because they felt at high risk, nor because they wanted to become healthier, as was hypothesised.

### Barriers and facilitators to continuation

#### Building a relationship

Almost all participants emphasised the importance of the relationship with the practice nurse:

*‘* [The nurses] *did their job very well.* […] *Not hurried but still goal-oriented and you can also talk about other things and get them off your mind. I can ask them anything. And I also get a decent answer. It’s excellent.’*

(P2, female, 77 years, living alone, ongoing participation)

The practice nurse was viewed as a dedicated person with up-to-date medical knowledge and a sincere interest in their personal circumstances.

Participants hoped for someone who was genuinely concerned about them and their health. If this turned out to be the case, they often felt very attached to their nurse. A long-standing relationship was necessary to gain trust and, ultimately, to make changes regarding their health:

*‘In this way* [by frequent visits to the same nurse], *you get an open relationship. For example, I can tell her everything. With others you keep more distance. But with her I know exactly where I stand as she does with me. She tries to do the right thing for me, that’s how I see it.’*

(P14, male, 78 years, living alone, ongoing participation)

Reiterating the importance of the relationship, those who had decided to quit the study reported that their nurse’s approach had prompted them to do so. They felt that the intervention could have been of more value to them, but they were disappointed by the impersonal, one-way communication, and lack of detailed questioning:

‘I hoped for more personal questions, more attention and personal contact. They should be much more people-oriented if they want to keep people involved.’

(P13, female, 79 years, living with partner, discontinued participation)

In some cases, participants noticed that the development of a personal bond was not feasible, due to frequent changes of nursing staff and/or the nurse being too young or inexperienced. For example, in one healthcare centre, two participants (P7 and P12) reported that no permanent nurse was available for a period of time. One also pointed out about the relatively inexperienced substitute nurses:

*‘Those youngsters, they might be nice people but not someone to begin a conversation with. Especially not when you’re past 75* [years of age]. *No experience of life.* […] *The difference with* [the former nurse] *was huge; she was in really close contact with you.’*

(P12, female, 81 years, living alone, discontinued participation)

For most participants the bond with the nurse, or its absence, was the main reason for continuing or quitting the programme, regardless of the identified risks or perceived benefits. Next to this personal bond, the approach of the nurse appeared to be critical.

#### Guarding autonomy

In general, participants wished to be involved in medical decisions, such as starting medication or getting additional diagnostic tests, but they were prepared to follow the advice of their health professional, even if they did not always fully understand the rationale:

*‘If it’s reasonable, of course, I accept it from her*. […] *If it is medically safe and they advise it, then I’ll take it. When the wise people say: “it’s better for you” well, they know better than me, so I just take the pills.’*

(P14, male, 78 years, living alone, ongoing participation)

For lifestyle issues however, participants all felt this was, and should be, entirely their own decision:

‘To live healthily, you do it or you don’t for yourself, that’s up to you.’

(P11, male, 82 years, living with partner, ongoing participation)

A number of interviewees expressed the importance of their autonomy being respected. They felt this became more pertinent with increasing age. Some interviewees clearly indicated that they were not prepared to follow any advice on specific domains at all. As one woman put it:

‘Actually, I have this point of view. I absolutely hate sports and such matters. So I will not do it. I’ll probably live for a few less years: so what.’

(P2, female, 77 years, living alone, ongoing participation)

There were similar examples for not eating breakfast, for smoking, and drinking alcohol, habits often developed several decades ago, to which participants had resigned themselves. Continued efforts to change these behaviours by nurses who were unaware of the underlying views or convictions caused resistance.

The term ‘lifestyle advice’ was generally regarded as patronising and comprised ‘things that one already knows’ (P2, P7) or things that were not relevant to the participant. Some participants felt irritated when lifestyle advice was given:

*‘I know what a healthy diet is. Not that I always do what’s best for my health, but I do know what it is.* […] *There was no advice at all that was useful to me. They were all things that I already knew. Lifestyle advice, I think that’s meddlesome. But that’s unkind to say.’*

(P7, male, 77 years, living alone, discontinued participation)

Another ex-participant felt disrespected and disregarded in the consultations in which the nurse gave advice. She had clear ideas about what approach should be used:

*‘ “You have to …”, always “you have to ...”; I detest it. They don’t ask you what you want to do about it yourself. I think it’s a bit offensive for elderly people. They* [the nurses] *are kind, you know, but it’s just, you get older and age is a heavy burden. They should use another method actually, I think. More um … ask more questions about your constitution, how you are doing and what you can do as an older person in your home etc. And that’s often forgotten.’*

(P13, female, 79 years, living with partner, discontinued participation)

Participants, therefore, wanted to be heard and respected, and able to discuss issues with the nurse as equal partners.

#### The coaching attitude

In contrast with the above, satisfied participants reported that they had not been advised on lifestyle and thought of this as something positive. For example, when asked ‘did the nurse give you any lifestyle advice?’, one participant answered:

‘No, no, I don’t think so. We did talk about certain things, but it was not advice but um … more like a conversation. What you can do to maintain your weight, those kind of things.’

(P3, female, 77 years, living with partner, ongoing participation)

The aversion to lifestyle advice seemed to be caused by its directive and moral nature; participants felt judged, rather than encouraged to change their behaviour. However, people generally appreciated the nurse enquiring about specific lifestyle issues and their efforts towards healthier living:

‘She asks about it, about physical activity and also ‘do you do household chores’, and if so ‘do you need help with it’ and um … because with household chores, you are being physically active too, you know. She also asks if you go walking and go outside. I don’t go cycling anymore, but I do have a home trainer, I tell her, and I use it. That’s also good, she says.’

(P10, female, 80 years, living with partner, ongoing participation)

Another woman illustrated the reflective way in which the nurse encouraged her to improve her diet:

*‘She never tells us* [my daughter and me] *what to do, not at all. She likes it when I tell her, that’s why she’s so good. We live healthier now. We eat two pieces of fruit every day, she always asks about it. Yes, we eat very healthy as a matter of fact.’*

(P1, female, 77 years, living with daughter, ongoing participation)

The positive effect of the coaching attitude described by participants was threefold:

- when the nurse asked questions and listened, they gained trust;
- nurses could recognise and reinforce efforts that had already been made; and, as a result; and
- they were able to discuss tangible, tailor-made plans with the participant.

Ironically, in accordance with the above, participants who succeeded in making (generally small) changes reported that the nurse had been of no, or only minor, influence: they felt it had been primarily their own decision to change their behaviour. For instance, one participant started cooking with oil instead of butter and, when asked why, he said hesitantly:

*‘Well, because of those conversations* [with the nurse] *probably, but I heard it from outside too, of course. And it’s also easier than before to get cooking oils in the shops. It used to be normal to use butter.* […] *It* [the influence of the nurse] *was only indirect, because I knew it already. No, the conversations were not the main reason* [for a change in lifestyle]*. I know we talked about it, and I took note of it, but I knew it already.’*

(P7, male, 77 years, living alone, discontinued participation)

It seemed that, with regard to making changes, especially in lifestyle, successful nurses operated in a sharply defined area; they mainly asked questions and discussed issues in an open conversation with participants, but stayed away from giving directions or general advice. In this way, participants felt heard and respected, and were more likely to stay engaged in the consultations.

## DISCUSSION

### Summary

Three main themes concerning participation in the cardiovascular prevention programme were identified. First, the perception of being ‘checked up on’ gave a sense of control, safety or being looked after. It was seen as an essential component of the programme, and a reason for starting and continuing participation.

Secondly, the personal approach of the practice nurse appeared to be crucial. Next to the nurse’s medical expertise, their ability to listen and to build a personal relationship was strongly linked to trust and prolonged participation.

Thirdly, participants wanted nurses to have a coaching and supporting attitude; they wanted to be actively involved in concerns about their health and be able to understand and negotiate both the intricacies and possibilities for change. Although practical help on how to implement changes was much appreciated, general preventive advice was regarded as unnecessary and patronising. Scheduled follow-ups were deemed necessary to build a relationship and to evaluate medication and lifestyle, along with any changes that had been made.

### Strengths and limitations

This study was carried out in the homes of older people living in the community by researchers who were independent of the GP practices; as such, the intervention enabled participants to feel relaxed and to speak freely about their experiences. In addition, a diverse population of older people was interviewed, and all had participated in the programme for several years. Non-white older people were under-represented in this study (mainly because of language barriers), which is a potential limitation. Some were very aware of the objective of the study, while others did not remember this, or even regarded the programme as usual care.

A number of interviewees had withdrawn from the trial, which gave a balanced view on the themes identified. As an example, ex-participants stated that they missed personal contact with the nurse, while this was spontaneously reported as a main strength by those who had not withdrawn. Although working with the same study protocol, nurses (mostly permanent employees of the GP practices) used different approaches when delivering care. This seems to increase the external validity of the results, as the nurses' working methods are representative of regular nurse-led care for chronic diseases.

A representative view was not aimed for; instead the aim was to capture the particular experiences of those participating in the programme. Nevertheless, it should be noted that participants who stopped shortly after the start of the study, or those who decided to not participate at all, were not interviewed. This allowed for an in-depth analysis of experiences of the programme and its strengths, but gives limited information of the views of the older population in general. Caution should, therefore, be taken when translating the results to other settings.

Interviewees participated mainly with their own health in mind, or because they were asked to do so by their GP. However, many spontaneously reported the importance of the research project in which they participated and, particularly, the topics of dementia and ageing in general were mentioned. Also, being able to do something for society and still being useful were highlighted. Altruism as a motivator could be indicative of an over-representation of relatively healthy and highly educated people in the research setting.^16^ This could be regarded as a limitation, because those who could possibly benefit most from the intervention might be under-represented.

### Comparison with existing literature

To the authors' knowledge, little is known about older people’s views and experiences of long-term, community-targeted, preventive initiatives. Based on limited evidence, a Cochrane review suggested that coaching could be used to improve the involvement of older people in primary care.^17^ In line with the current findings, a study on the cognitive and emotional effects of preventive consultations showed that insufficient tailoring to one’s personal situation negatively influenced the perceived benefits of an intervention.^18^

In a lifestyle programme promoting physical activity in a deprived community, discussing personal and social issues with the nurse was regarded as a main benefit of the programme.^19^ Two studies on cardiovascular prevention reported that participants felt that they had sufficient knowledge on how to reduce cardiovascular risk, but appreciated a hands-on intervention programme to help them put that knowledge into practice.^19^,^20^ This mirrors the findings of this study. A scheduled follow-up was considered necessary by many interviewees from both single and multiple consultation programmes in order to build a fruitful relationship between healthcare provider and participant, and to help maintain changes.^11^,^18^,^19^,^21^,^22^ Previous research has shown that regular follow-ups increase the likelihood of sustained behaviour change.^19^ In this study too, participants reported a more positive attitude to the programme when there had been regular follow-ups with the same nurse.

In several studies, a lack of professional help in changing lifestyle was identified and more training of the healthcare provider (GP or nurse) to improve counselling skills has often been advised.^11^,^19^,^23^–^25^ Nowadays, numerous strategies are available for this purpose.^26^ Over the past years, many, but not all, participating practice nurses followed training in motivational interviewing outside the framework of this study. This counselling approach has been shown to outperform the provision of traditional advice in 80% of studies.^27^ Although motivational interviewing itself was not investigated here, its elements can be recognised throughout the analysis and may have contributed to the success of the intervention.

The actual effect of the nurse-led intervention could not be verified in this study, however, nurses seem to be able to positively influence aspects of a healthy lifestyle by coaching their participants. It is conceivable that their efforts result in better outcomes through sustained healthy behaviours, better medication adherence, and small improvements in lifestyle. This idea is supported by a systematic review on complex, preventive interventions for older people, which showed reduced nursing home and hospital admissions.^28^ A recent trial reported a decreased incidence of long-term care dependency after a comparable intervention by GPs.^2^

### Implications for practice

Long-term cardiovascular prevention consultations are appreciated by older people when carried out by a dedicated nurse, who is able to integrate care with the personal needs and beliefs of participants. Key elements for successful implementation, with respect to participant satisfaction and adherence, are to build a long-term, personal relationship and to adopt a coaching approach, with regular follow-ups. General lifestyle advice, often thought to be a crucial element of preventive consultations, was regarded as unnecessary, and even patronising, by participants; instead, the strength of the approach appeared to be to listen to the participant and coach them, dealing with concrete examples or possibilities, rather than general ideals of healthy living.
